# Classification of Alzheimer’s Disease Based on Deep Learning of Brain Structural and Metabolic Data

**DOI:** 10.3389/fnagi.2022.927217

**Published:** 2022-07-12

**Authors:** Huiquan Wang, Tianzi Feng, Zhe Zhao, Xue Bai, Guang Han, Jinhai Wang, Zongrui Dai, Rong Wang, Weibiao Zhao, Fuxin Ren, Fei Gao

**Affiliations:** ^1^School of Life Sciences, Tiangong University, Tianjin, China; ^2^School of Electrical and Information Engineering, Tiangong University, Tianjin, China; ^3^Department of Radiology, Qilu Hospital of Shandong University, Jinan, China; ^4^Westa College, Southwest University, Chongqing, China; ^5^Department of Radiology, Shandong Provincial Hospital Affiliated to Shandong First Medical University, Jinan, China

**Keywords:** Alzheimer’s disease, deep learning, magnetic resonance imaging, magnetic resonance spectroscopy, feature extraction

## Abstract

To improve the diagnosis and classification of Alzheimer’s disease (AD), a modeling method is proposed based on the combining magnetic resonance images (MRI) brain structural data with metabolite levels of the frontal and parietal regions. First, multi-atlas brain segmentation technology based on T1-weighted images and edited magnetic resonance spectroscopy (MRS) were used to extract data of 279 brain regions and levels of 12 metabolites from regions of interest (ROIs) in the frontal and parietal regions. The *t*-test combined with false discovery rate (FDR) correction was used to reduce the dimensionality in the data, and MRI structural data of 54 brain regions and levels of 4 metabolites that obviously correlated with AD were screened out. Lastly, the stacked auto-encoder neural network (SAE) was used to classify AD and healthy controls (HCs), which judged the effect of classification method by fivefold cross validation. The results indicated that the mean accuracy of the five experimental model increased from 96 to 100%, the AUC value increased from 0.97 to 1, specificity increased from 90 to 100%, and F1 value increased from 0.97 to 1. Comparing the effect of each metabolite on model performance revealed that the gamma-aminobutyric acid (GABA) + levels in the parietal region resulted in the most significant improvement in model performance, with the accuracy rate increasing from 96 to 98%, the AUC value increased from 0.97 to 0.99 and the specificity increasing from 90 to 95%. Moreover, the GABA + levels in the parietal region was significantly correlated with Mini Mental State Examination (MMSE) scores of patients with AD (*r* = 0.627), and the F statistics were largest (*F* = 25.538), which supports the hypothesis that dysfunctional GABAergic system play an important role in the pathogenesis of AD. Overall, our findings support that a comprehensive method that combines MRI structural and metabolic data of brain regions can improve model classification efficiency of AD.

## Introduction

AlzheimerIONtisease (AD) is a progressive neurodegenerative disease that mainly manifests as cognitive decline and abnormal behavior (Zeng et al., 2021). The onset of AD is hidden, and thus it is difficult to identify in the early stages. However, as AD progresses, it seriously affects daily life and causes irreversible damage to the brain, resulting in a heavy burden on the patient’s family and the healthcare system. At present, there is no effective clinical method to prevent or treat AD, and existing drugs are only able to slow down disease progression. As a result, early diagnosis of AD has become one of the biggest challenges currently facing medicine and society (Reitz and Mayeux, 2014; Vs et al., 2020). Magnetic resonance imaging (MRI), as the main method of neuroimaging, has been widely used to study the structure, function, and perfusion of the human brain. Notably, structural MRI is widely used in the early diagnosis of AD (Konrad et al., 2009). Previous studies have shown that cortical thickness and volume of hippocampus play a pivotal role in the classification between AD and healthy controls (Liu et al., 2011).

However, functional changes in AD occurs earlier than structural changes, i.e., AD patients show abnormal brain function in the early stage before impairment of brain structure has occurred (Bosco et al., 2017; Kamagata et al., 2020). For instance, a resting-state functional MRI study suggested that the activity in the default mode network may prove a sensitive and specific biomarker for early stage AD (Md et al., 2004; Wang et al., 2012).

Therefore, adopting structural MRI alone for the diagnosis of early AD has major limitations and its specificity and sensitivity are low. Importantly, previous studies have shown that AD also involves dysfunction of a variety of neurotransmitter systems, including the cholinergic system, glutamatergic (Glu) system, and gamma-aminobutyric acid (GABA) system (Zhang et al., 2015). Magnetic resonance spectroscopy (Munteanu et al., 2015; Vignoli et al., 2020) (MRS), which is a non-invasive technology that uses the principle of chemical shift to measure metabolite levels in the brain, has been widely used in studies of various neurological and mental diseases. For example, N-acetylaspartate (NAA) can reflect damage and loss of neuronal cells in brain tissue. Kantarci et al. (2007) and Carelli et al. (2015) found that NAA levels in the hippocampus can be used as an imaging marker to assess the progression of mild cognitive impairment to AD. GABA is an important inhibitory neurotransmitter (Jo et al., 2014) that regulates excitatory activity, thereby preventing overexcitement of neurons and oscillation activity of neural networks. Studies of AD animal models have indicated that abnormal function of the GABAergic system may be a common target of multiple abnormal signal pathways in AD (Danlei et al., 2020). Furthermore, abnormal function of the GABAergic system can lead to neuronal excitation-inhibition imbalance, which promotes the pathological spread of Aβ and tau and further aggravates cognitive impairment in AD patients. Additionally, studies of post-mortem brains have reported decreased GABA levels in the frontal and parietal brain tissues of AD patients (Kreisl et al., 2013; Salminen et al., 2016). The MEGA-PRESS (MEscher-GArwood-Point RESolved Spectroscopy) sequence has been applied to detect brain GABA levels in many neurologic and psychiatric disorders (Atagün et al., 2017; Murley et al., 2020; He et al., 2021).

However, only a few studies to date have combined structural data and metabolite levels of brain regions to establish an early diagnosis model of AD. (Thus, this study adopted multi-atlas segmentation technology based on high-resolution brain structure to automatically segment the brain to extract the structural data and then applied MEGA-PRESS sequence to detect the levels of metabolites.

With advances in artificial intelligence, deep learning has demonstrated a good classification effect for the diagnosis of AD (Ortiz et al., 2016; Suk et al., 2017; Liu et al., 2020). The convolutional neural network (CNN) (Abdulazeem et al., 2021) is often used as a modeling and classification tool to diagnose AD; however, CNN needs updating of several parameters within its network. With the increasing of hidden layers, CNN network will become complex, adding a lot of computing process. Stacked auto-encoder (SAE) (Suk et al., 2015; Ijjina and Mohan, 2016) is a deep learning network model for unsupervised learning that is widely used in various feature extraction and classification problems. SAE neural network has the function of feature selection, which can find the features of interest more easily (Cao et al., 2016). Combining with dense layer, SAE can used to perform supervised learning by achieving the approximation of complex function. The SAE neural network used in this study encodes the input data to obtain data characteristics and then decodes the transmitted data to realize the reconstruction of the input data. Its structure is relatively simple, the reconstruction speed is fast, and there is no requirement for a large sample size. In the present study, the SAE neural network was used to perform AD classification based on different types of input data, namely MRI structural data of brain regions and MRS metabolite levels of frontal and parietal regions. Moreover, the accuracy, the area under the curve (AUC) value, sensitivity, and specificity of the AD classification models were calculated in order to evaluate the impact of the addition of MRS metabolic data on the accuracy of the AD classification results.

## Materials and Methods

### Study Design

This study was approved by the ethics committee of Shandong First Medical University and all participants or their legal guardians provided written informed consent. In total, 27 AD patients and 15 age-, sex-, and education-level matched healthy controls (HCs) were included in this study. AD patients were diagnosed according to the National Institute of Neurological and Communicative Disorders and the Alzheimersityisease and Related Disorders Association criteria (NINCDS-ADRDA) (Dubois et al., 2007). The AD group comprised 11 men and 16 women with an average age of 65.95 ± 6.28 years and a mean Mini Mental State Examination Scale (MMSE) (Davatzikos et al., 2011) score of 18.78 ± 2.91. The HCs group comprised 8 males and 7 females with an average age of 63.87 ± 3.32 years and a mean MMSE score of 28.47 ± 0.96. The details are shown in Table 1. The exclusion criteria for the AD group were as follows: (1) It may be dementia other than AD, such as vascular dementia, paralytic dementia, dementia syndrome caused by other brain or physical diseases, etc. (2) Those who are unable to cooperate with severely impaired speech expression, comprehension, severe visual and hearing impairment, and other reasons. The exclusion criteria for the AD and HCs group were as follows: (1) MRI contraindications. (2) A history of substance abuse.

**TABLE 1:** Participants’ demographic and clinical information.

Characteristics	AD group	HC group	*P*-value
Subject	27	15	–
Gender (M/F)	11/16	8/7	0.59
Age	67.11 ± 7.18	63.87 ± 3.32	0.11
MMSE	18.78 ± 2.91	28.47 ± 0.96	<0.001

*The data are presented as means ± standard deviations. AD, Alzheimer’s disease; HC, health controls.*

### Data Acquisition

MRI data were acquired using the dual radio frequency transmission mode of a Philips 3.0T scanner (Achieva, TX) and 8-channel head coils as transmitting and receiving coils. The T1-weighted TFE sequence was used to obtain high-resolution 3D brain images. The scan related parameters were: pixel size = 1 × 1 × 1 mm^3^, matrix = 256 × 256, thickness = 1 mm, repetition time (TR) = 8.2 ms, echo time (TE) = 3.7 ms, reversal angle = 8°, and field of view (FOV) = 24 × 24 cm^2^. On the 3D T1-TFE image, the regions of interest (ROIs) were located in the frontal and parietal regions with a size of 3 × 3 × 3 cm^3^. The frontal region was placed above the anterior half of the corpus callosum and parallel to the corpus callosum. The parietal region was placed in the middle region of the bilateral parietal region above the corpus callosum and parallel to the tangent line of the corpus callosum. The edges of all ROIs avoided touching the skull and bilateral lateral ventricles.

The MEGA-PRESS sequence was used for metabolite data collection. The scan parameters were as follows: scan time = 11 min, TR = 2,000 ms, TE = 68 ms, scan bandwidth = 1,000 Hz, and 320 averages. LCmodel software was used to quantify the levels of metabolite in two ROIs. Since MEGA-PRESS technology obtains the GABA signal at 3.02 ppm, it also contains the signals of macromolecules and high carnosine, so the collected signal is GABA + rather than pure GABA.

### *T*-test Analysis

In order to establish an accurate AD classification model, it is first necessary to screen out MRI structural data of brain regions and metabolite levels of ROIs that are closely related to AD. We used the independent samples *t*-test for multi-modal data analysis to achieve effective dimensionality reduction. The dataset comprising multi-modal data was used as the input data of the classification model, and the AD disease label was used as the target of the classification model. The multi-modal data included MRI structural data of brain regions and the levels of various metabolites in the frontal and parietal regions. The AD disease label is represented by 1 and 0, where 1 represents AD and 0 represents HCs. An FDR corrected *p*-value < 0.05 suggests that the multimodal data correlate with the target of the AD classification model, indicating that the difference is statistically significant (Hidalgo-Muñoz et al., 2014).

### Stacked Auto-Encoder Neural Network Modeling Method

#### Stacked Auto-Encoder Network Model

**FIGURE 1:**
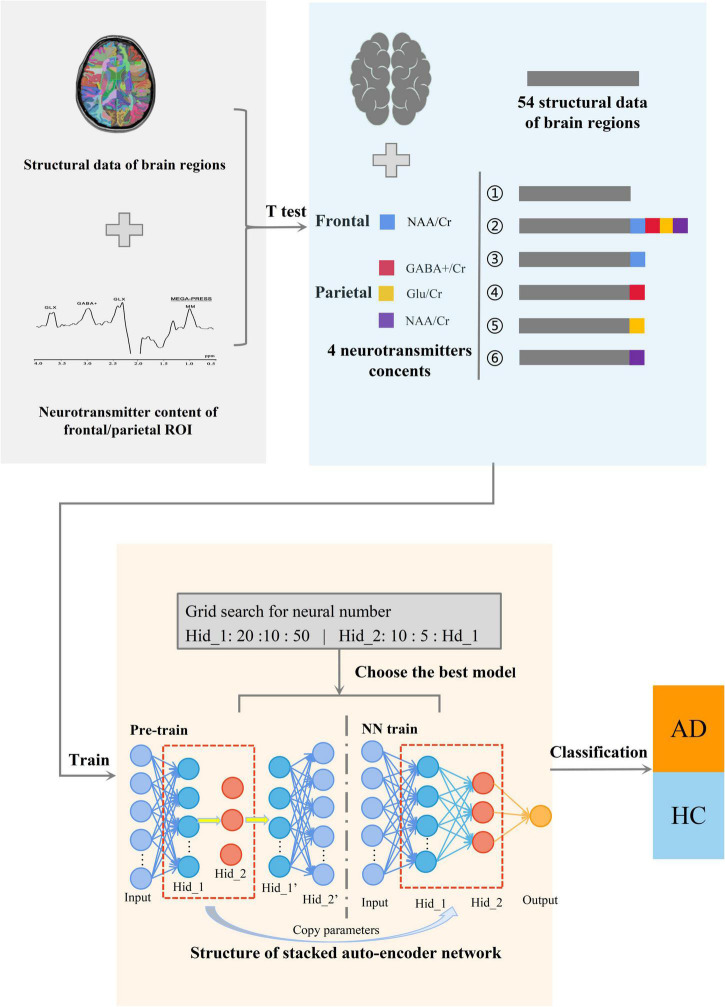
Diagram of the overall structure of the classification model. The whole is divided into three parts, from left to right, the first part is to collect the structural data of brain regions and metabolite levels of the ROI in the frontal and parietal region. The second part is to carry out multi-model data processing of the obtained data. And the third part is to classify AD using SAE neural network.

The SAE neural network is a deep neural network model for unsupervised feature recognition that is widely used in various feature extraction and classification problems. To achieve accurate classification of AD, this study uses a deep neural network composed of double hidden layer auto-encoders (AE). Among them, AE is a neural network with a three-layer structure comprising an input layer, hidden layer, and output layer. Each layer is fully connected. The target of SAE is to estimate the input data accurately while filtering the unnecessary information. By achieving this target, the encoder layers have lower dimensions than the original data, reducing the redundant information. Then, decoder layers are used to regenerate the feature map based on the encoder layers, gaining a novel dataset. The SAE neural network in this study is composed of one SAE model and one classification layers that includes two stages: unsupervised pre-training and supervised fine-tuning. The first stage pre-trains the AE of the SAE neural network in an unsupervised manner using a layer-by-layer greedy algorithm, i.e., an algorithm that uses the feature weights of the previous layer of the AE as the input data of the next layer in the order of front to back. Pre-training is performed layer by layer. In the second stage, add the label corresponding to the sample of this study and the weight value obtained in the previous stage, and use the MSE loss function to calculate the error between the predicted value and the real value. Then, according to the derivative of the loss function, the error is transmitted back along the minimum direction of the gradient to correct each weight value in the forward calculation formula. Finally, the whole training process of SAE neural network is completed. As shown in Figure 1, the overall structure of the AD classification model used in this study includes three main parts: (i) screening key data using the *t*-test and combining input datasets, (ii) using deep learning to train the classification model, and (iii) selecting the best model and classifying AD and HC.

**FIGURE 2:**
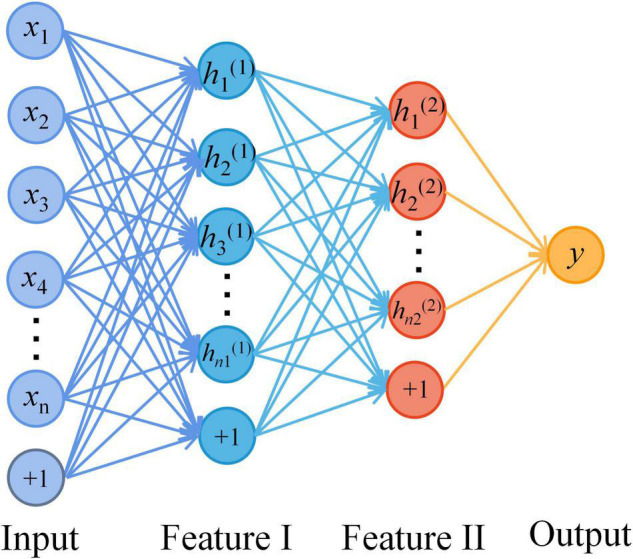
The structure diagram of the stacked auto-encoder (SAE) neural network. A four-layer SAE neural network with double hidden layers was selected for AD classification. The neurons of all layers are connected in a fully connected way. Among them, the number of the input layer neurons is the dimensions of multimodal data. The number of hidden layer neurons is set by the method of selecting the best model. And the output layer is AD or HC.

Figure 2 shows the SAE neural network structure. The input layer is the multi-modal structural data of brain regions and the metabolite levels of the frontal and parietal regions. The output layer is the AD classifier of 0 or 1. In this study, SAE neural network is also used for feature selection of input features. The number of neurons in the hidden layers are optimized by using the grid search method. First, in the Hidden_1, searching starts at 10, with a step length of 10 and ends at 50. Then, keeping the number of Hidden_1 layer unchanged, altering the number of Hidden_2 layer with the sequence starting at 10 and ends at the number of neurons equal Hidden_1 by step size of 5. Based on the above calculations, the results of the traversal are used to select the best model. It is determined that the good SAE neural network has better sparsity, so as to avoid the dimension curse problem that may occur when the number of features is greater than the number of samples in the process of network training. In the pre-training stage, the learning rate of the model is set to 0.1, the batch size is set to 3, and the epochs are related to the number of training sets. In the formal training stage, the learning rate of the model is 1, the batch size is related to the number of training sets, the epochs are 15, and the activation function in the classification layer is the Sigmoid function.

#### Input Dataset Classification

In this study, in order to ensure the generalization ability of the constructed AD classification model and avoid over fitting of the model, the K-fold cross-validation method with K equal to 5 was selected to test the performance of the classification model. The 42 groups of sample datasets were divided into five parts, which take four of them as the training set to train the classification model each time and used the remaining one as the test set to judge the performance of the classification model. The experimental steps were repeated for five times. And the constructed AD classification model was evaluated by the overall results of the prediction set.

## Results

### *T*-test Screening Results

Based on the *t*-test and FDR data correction results, a total of 58 multi-modal data features showing significant differences between groups were obtained (Table 2), including data for 54 MRI structural data of brain regions and 4 levels of metabolites.

**TABLE 2:** Data screened using the *t*-test.

Brain region	*P*-value	Brain region	*P*-value
Inferior frontal WM pars opercularis_R	0.0478	Cingulum (cingulate gyrus)_L	0.0199
Inferior frontal WM pars orbitralis_R	0.0455	Putamen_L	0.0199
Superior frontal gyrus_L	0.0418	Middle Temporal WM_L	0.0187
posterior cingulate gyrus_R	0.0400	Superior corona radiata_L	0.0187
Angular gyrus_R	0.0387	Nucleus accumbens_R	0.0187
Insula_L	0.0387	Fusiform Gyrus_R	0.0163
NAA/Cr_F	0.0379	Fimbria_L	0.0162
Parahippocampal gyrus_L	0.0363	Hippocampus_R	0.0162
Superior temporal gyrus_R	0.0356	Lateral Fronto-Orbital WM_R	0.0162
Superior parietal gyrus_L	0.0354	Posterior Cingulate WM_L	0.0162
Splenium of corpus callosum_L	0.0341	Middle Temporal Gyrus_R	0.0162
Middle frontal gyrus (posterior segment)_L	0.0342	Temporal Lobe Sulci_R	0.0153
Inferior fronto-occipital fasciculus_R	0.0342	Inferior Frontal WM pars opercularis_L	0.0109
Cingulum (hippocampus)_L	0.0330	Dorsal anterior cingulate gyrus_L	0.0109
Body of corpus callosum_L	0.0305	Middle Temporal Gyrus_L	0.0103
Substancia Nigra_R	0.0305	Inferior frontal gyrus pars opercularis_L	0.0103
External capsule_R	0.0294	Nucleus accumbens_L	0.0097
Fusifrom gyrus_L	0.0294	Clustrum Complex_L	0.0095
Supramarginal gyrus_R	0.0290	BasalForebrain_R	0.0084
Superior temporal gyrus_L	0.0273	Sylvian Fissure Temporal Lobe Part_L	0.0076
Pole of middle temporal gyrus_L	0.0253	Amygdala_R	0.0076
Anterior corona radiata_R	0.0235	Inferior Frontal WM pars Triangularis_L	0.0055
BasalForebrain_R	0.0235	Sylvian Fissure Frontal Lobe Part_L	0.0041
Occipital Lobe Sulci_R	0.0235	Superior longitudinal fasciculus_L	0.0035
Subcallosal anterior cingulate WM_L	0.0235	Amygdala_L	0.0031
Inferior temporal gyrus_L	0.0231	NAA/Cr_P	0.0020
GABA + /Cr_P	0.0206	Caudate_tail_L	0.0020
Superior corona radiata_R	0.0199	Glu/Cr_P	0.0003
Middle Frontal WM (posterior segment)_L	0.0199	Hippocampus_L	0.0003

*FDR corrected p < 0.05. L, left; R, right; F, frontal region; P, parietal region. WM, white matter.*

**FIGURE 3:**
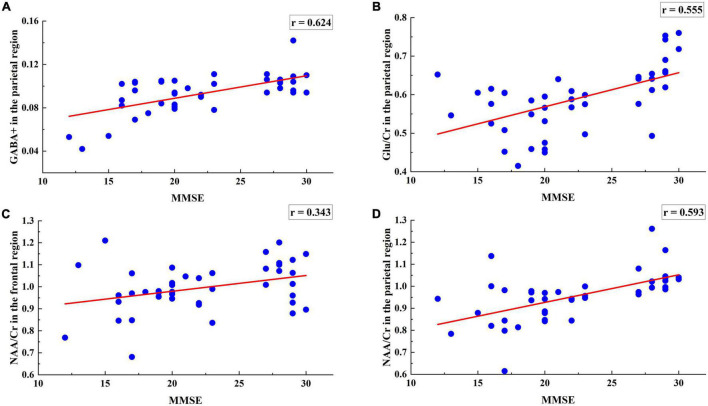
Regression analysis of 4 metabolites including the parietal region GABA +, Glu/Cr, NAA/Cr levels and the frontal region NAA/Cr levels with MMSE. **(A)** MMSE scores were positively associated with the GABA + levels of the parietal region (*r* = 0.624, *p* = 0.0165). **(B)** MMSE scores were positively associated with the Glu/Cr levels of the parietal region (*r* = 0.095, *p* = 0.0068). **(C)** MMSE scores were negatively associated with the NAA/Cr levels of the frontal region (*r* = −0.155, *p* = 0.0412). **(D)** MMSE scores were positively associated with the NAA/Cr levels of the parietal region (*r* = 0.360, *p* = 0.000002).

To further verify whether these 58 multi-modal features are consistent with AD diagnosis, the correlation analysis was performed for each feature and the MMSE scores. Using the multi-modal data as the independent variable and the MMSE scores as the dependent variable, the r correlation coefficient was calculated to further verify the reliability of data selection. Figures 3A–D shows the regression results for the 4 metabolite levels and MMSE scores.

The range of the correlation *r*-values is (0.343, 0.694), indicating that the selected metabolite data have a strong correlation with MMSE and supporting that the selected 4 metabolites in the frontal and parietal regions correlated with AD symptoms.

### Input Data Classification of Stacked Auto-Encoder Neural Network

As shown in Table 3, combining the multi-modal data according to the screening results resulted in 7 different AD classification models, respectively, labeled ➀–➆. The input dataset is multi-modal data composed of MRI structural data of brain regions and metabolites in different ROIs. The output dataset is the presence of AD (0 = HC, 1 = AD). Model ➀ includes the control group and the input dataset is the 54 MRI structural data of brain regions selected by the *t*-test. Model ➁ includes the 54 MRI structural data of brain regions combined with the 4 metabolites, including NAA/Cr in the frontal region and GABA +, Glu/Cr and NAA/Cr in the parietal region. Models ➂–➅ are the 4 input datasets obtained by combining the 54 structural of brain regions and the data for each metabolite selected by the *t*-test. Model ➆ includes the 54 MRI structural data of brain regions combined with GABA +, Glu/Cr and NAA/Cr in the parietal region. The fivefold cross validation results of the seven models are shown in Supplementary Tables 1–7.

**TABLE 3:** Input datasets of each classification model.

Parameter combination	
**➀**	54 Structural data
**➁**	54 Structural data + 4 Metabolite levels data
**➂**	54 Structural data + GABA + in the parietal region
**➃**	54 Structural data + Glu/Cr in the parietal region
**➄**	54 Structural data + NAA/Cr in the frontal region
**➅**	54 Structural data + NAA/Cr in the parietal region
**➆**	54 Structural data + 3 Metabolic Data in the parietal region

*4 Metabolite Levels Data include GABA +, Glu/Cr and NAA/Cr in the parietal region and NAA/Cr in the frontal region.*

### Comparison of the Alzheimer’s Disease Classification Accuracy of Different Models

**FIGURE 4:**
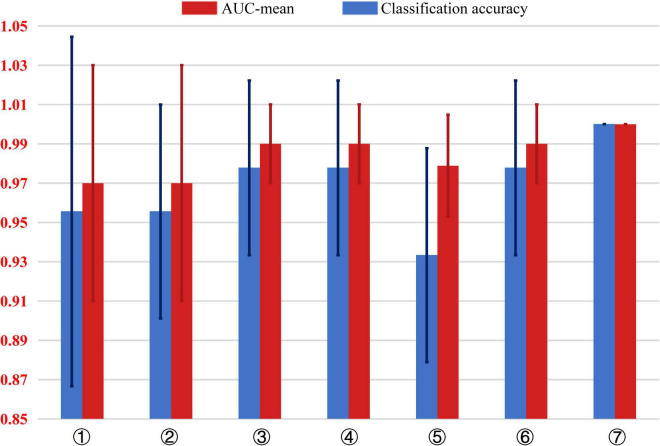
Comparison of the classification accuracy and AUC value of 7 different AD classification models. The input dataset of model ➀ are 54 structural data. The input dataset of model ➁ are 54 structural data and 4 metabolite levels data including GABA +, Glu/Cr, NAA/Cr in the parietal region and NAA/Cr in the frontal region. The input dataset of model ➂ are 54 structural data and GABA + in the parietal region. The input dataset of model ➃ are 54 structural data and Glu/Cr in the parietal region. The input dataset of model ➄ are 54 structural data and NAA/Cr in the frontal region. The input dataset of model ➅ are 54 structural data and NAA/Cr in the parietal region. The input dataset of model ➆ are 54 structural data and GABA +, Glu/Cr and NAA/Cr in the parietal region.

In the study, the mean of the five experimental results in the fivefold cross-validation of each model was taken as the final experimental result. Figure 4 presents an intuitive comparison chart of the classification accuracy and AUC values of the 7 models.

As shown in Figure 4, comparing model ➀, ➁, and ➆, adding the three metabolites of GABA+, Glu/Cr, and NAA/Cr in the parietal region increases the accuracy of AD classification from 96 to 100%, The AUC value from 0.97 to 1, indicating significant improvement in the classification performance.

Models ➂–➅ show the combinations of one of the 4 metabolites with the 54 MRI structural data of brain regions. Compared with model ➀ that includes only structural data, the AD classification accuracy of model ➂, ➃, and ➄ with GABA +, Glu/Cr, and NAA/Cr in the parietal region, respectively, increased from 6 to 98%, and the AUC value was improved from 0.97 to 1.00. However, the AUC value of model ➄ combined with NAA/Cr in the frontal region and brain structural data increased from 0.97 to 0.98, and the classification accuracy decreased from 97 to 82%.

From the results of linear regression between the metabolite data and MMSE in Table 4, the four metabolite data of GABA+, Glu/Cr, NAA/Cr in the parietal region and NAA/Cr in the frontal region had significant statistical significance with AD classification (*p* < 0.05). Moreover, the four regression models passed the *F*-test, and the GABA + in the parietal region and MMSE regression models had the largest F statistics (*F* = 25.538). The results of the significance test further indicated that GABA + in the parietal region was the key data affecting AD classification.

**TABLE 4:** Four metabolite data in the parietal and frontal regions of ROI and MMSE linear regression analysis.

	*p*	Person’s r	R square	Adjusted R square	*F* statistic
GABA + _P	0.000010	0.624	0.390	0.374	25.538
Glu/Cr_P	0.000137	0.555	0.308	0.291	17.798
NAA/Cr_F	0.026000	0.343	0.118	0.095	5.329
NAA/Cr_P	0.000035	0.593	0.351	0.335	21.680

*p, p-value of significance; F, in the frontal region; P, in the parietal region.*

## Discussion

The results of this study show that the addition of the three metabolites GABA +, Glu/Cr, and NAA/Cr in the parietal region significantly improved AD classification. And of the various metabolites tested, the GABA + levels of the parietal region contributed the most to the model classification results, suggesting that it is a key feature that affects AD classification.

Structural MRI data alone are widely used in the diagnosis of AD. For example, atrophy of the hippocampus and entorhinal region are characteristic structural changes of AD. Previous studies have shown that the sensitivity of an AD model containing only MRI structural features is 93% and the specificity is 86% (Westman et al., 2010). In this study, the AD classification accuracy of the model containing MRI structural data of brain regions is 96%, and the specificity is 90%. In addition to MRI structural data of brain regions, changes in metabolites have been shown to be effective biological markers of AD. In the study, the addition of all 4 metabolites increased the accuracy of AD classification from 96 to 100%, the AUC value from 0.97 to 1.00 and the specificity from 90 to 100%. Previous studies have used multivariate data analysis to explore the effect of adding metabolite data to AD diagnostic models, which found that adding metabolite characteristics to a structural diagnostic model increased sensitivity to 97% and specificity to 94% (Westman et al., 2010). Some researchers (Ahmed et al., 2020) have used deep learning to classify early AD patients and controls using MRS data. The addition of MRS data increased the model classification accuracy to 93.3% and the specificity to 89.47%, indicating great detection efficiency. The above findings are consistent with the results of the present study, indicating that an AD classification method that combines MRI structural data of brain regions with metabolite levels has higher specificity and classification accuracy than a structural model alone, which can effectively improve the accuracy of AD diagnosis.

This study also analyzed the effects of four metabolites on the classification of AD. Figure 3 shows that the correlation of GABA + in the parietal region is the highest and that of NAA/Cr in frontal region is the lowest. The results showed that GABA + levels in the parietal region were the key data affecting AD classification. The model including GABA + had the highest accuracy and specificity, which increased the classification accuracy from 96 to 98%, AUC value from 0.97 to 0.99 and the specificity from 90 to 95%. The significant *p*-value of NAA/Cr of the frontal region was between 0.01 and 0.05, which was statistically significant. The significant *p*-values of GABA +, Glu/Cr, and NAA/Cr in the parietal region were all less than 0.01, which had extremely significant statistical significance with MMSE. Moreover, the four regression models passed the *F*-test, and the GABA + levels of the parietal region and MMSE regression models had the largest F statistics (*F* = 25.538), which further indicated GABA + levels of the parietal region is the key data affecting AD classification. GABA, which is the main inhibitory metabolite in the human brain, is closely related to cognitive function and participates in the regulation of various advanced cognitive behaviors such as learning and memory (Schmitz et al., 2017; Scholl et al., 2017). Studies of AD animal models have found that abnormal function of the GABAergic system may be a common target of multiple abnormal signal pathways in AD. Dysfunctional GABAergic system leads to neuronal excitation-inhibition imbalance, which will promote the pathological spread of Aβ and tau and further aggravate cognitive impairment in AD patients (Danlei et al., 2020). Moreover, excitatory-inhibitory imbalance is considered an important cause of cognitive impairment, and GABAergic system is an important regulatory factor for the excitation-inhibition balance (Duncan et al., 2014). Decreased parietal GABA levels may lead to regional excitation-inhibition imbalance, which may result in neuronal overexcitation. Thus, selectively manipulating the synthesis or release of GABA to correct excitation-inhibition imbalance could be a potential treatment target for AD. Overall, our findings provide further evidence that parietal GABA level is a key feature affecting the classification of AD. Combined with MRI structural data and metabolite characteristics, the accuracy of AD classification will be significantly improved.

Although our findings support that the method proposed in this study has potential clinical applications in AD research, our study is still subject to some limitations. First, the sample comprised only 42 AD patients and controls, and small number of samples may slow the convergence speed of neural network, causing overfitting and low generalization capability. Second, our study lacked information about longitudinal changes of structural and metabolic data in patients with AD. Thus, future studies should expand the number of participants and explore the impact of longitudinal data on the classification of AD over time, and we will try to apply novel method in prediction. Such as applying RNN or LSTM to classify. Lastly, we did not collect brain function and perfusion data in this study, such as resting-state functional MRI and arterial spin labeling perfusion. We plan to add the above data to subsequent studies to establish an AD diagnostic model involving three dimensions, namely structure, function, and metabolism.

## Conclusion

In summary, compared to AD classification models that only involves MRI brain region structural data, the addition of the metabolite levels in the parietal regions significantly improved AD classification performances. Moreover, the GABA + levels of the parietal region were the key feature affecting AD classification, which supports the hypothesis that dysfunctional GABAergic system plays an important role in the pathogenesis of AD. Our findings support that a comprehensive method that combines MRI structural and metabolic data of brain regions can improve model classification efficiency of AD.

## Data Availability Statement

The datasets presented in this article are not readily available because the corresponding data may expose privacy. Requests to access the datasets should be directed to corresponding authors.

## Ethics Statement

This study was approved by the ethics committee of Shandong First Medical University and all participants or their legal guardians provided written informed consent. Written informed consent was obtained from the individual(s) for the publication of any potentially identifiable images or data included in this article.

## Author Contributions

FG, HW, ZZ, GH, and JW contributed to the conception and design of this study. FG and FR summarize the database. ZD and XB were statistically analyzed. HW and FG wrote the first draft of the manuscript. TF, RW, and WZ wrote various parts of the manuscript. All authors participated in the revision, reading, and approval of the submitted version of the manuscript.

## Conflict of Interest

The authors declare that the research was conducted in the absence of any commercial or financial relationships that could be construed as a potential conflict of interest.

## Publisher’s Note

All claims expressed in this article are solely those of the authors and do not necessarily represent those of their affiliated organizations, or those of the publisher, the editors and the reviewers. Any product that may be evaluated in this article, or claim that may be made by its manufacturer, is not guaranteed or endorsed by the publisher.
