# Supplementary material for: Classification of Alzheimer’s Disease Based on Deep Learning of Brain Structural and Metabolic Data
Source: Front Aging Neurosci. 2022 Jul 12;14:927217. doi: 10.3389/fnagi.2022.927217 (PMC9315355; doi:10.3389/fnagi.2022.927217)
Supplement: Supplementary file 1 [file Data_Sheet_1.docx]

Supplementary Material

**Supplementary table 1**

| 54 Structural Data | | | | | | |
| --- | --- | --- | --- | --- | --- | --- |
| Group | 1 | 2 | 3 | 4 | 5 | Mean(±Std) |
| ACC | 0.78 | 1.00 | 1.00 | 1.00 | 1.00 | 0.96(±0.09) |
| Sen | 1.00 | 1.00 | 1.00 | 1.00 | 1.00 | 1.00(±0.00) |
| Spe | 0.50 | 1.00 | 1.00 | 1.00 | 1.00 | 0.90(±0.20) |
| F1 | 0.83 | 1.00 | 1.00 | 1.00 | 1.00 | 0.97(±0.07) |
| AUC | 0.85 | 1.00 | 1.00 | 1.00 | 1.00 | 0.97(±0.06) |

**Supplementary table 2**

| 54Structural Data+ 4 Metabolite Levels Data | | | | | | |
| --- | --- | --- | --- | --- | --- | --- |
| Group | 1 | 2 | 3 | 4 | 5 | Mean(±Std) |
| ACC | 0.89 | 0.89 | 1.00 | 1.00 | 1.00 | 0.96(±0.05) |
| Sen | 1.00 | 1.00 | 1.00 | 1.00 | 1.00 | 1.00(±0.00) |
| Spe | 0.75 | 0.67 | 1.00 | 1.00 | 1.00 | 0.88(±0.15) |
| F1 | 0.91 | 0.92 | 1.00 | 1.00 | 1.00 | 0.97(±0.04) |
| AUC | 0.85 | 1.00 | 1.00 | 1.00 | 1.00 | 0.97(±0.06) |

**Supplementary table 3**

| 54 Structural Data + GABA+ in the parietal region | | | | | | |
| --- | --- | --- | --- | --- | --- | --- |
| Group | 1 | 2 | 3 | 4 | 5 | Mean(±Std) |
| ACC | 0.89 | 1.00 | 1.00 | 1.00 | 1.00 | 0.98(±0.04) |
| Sen | 1.00 | 1.00 | 1.00 | 1.00 | 1.00 | 1.00(±0.00) |
| Spe | 0.75 | 1.00 | 1.00 | 1.00 | 1.00 | 0.95(±0.10) |
| F1 | 0.91 | 1.00 | 1.00 | 1.00 | 1.00 | 0.98(±0.04) |
| AUC | 0.95 | 1.00 | 1.00 | 1.00 | 1.00 | 0.99(±0.02) |

**Supplementary table 4**

| 54 Structural Data + Glu/Cr in the parietal region | | | | | | |
| --- | --- | --- | --- | --- | --- | --- |
| Group | 1 | 2 | 3 | 4 | 5 | Mean(±Std) |
| ACC | 0.89 | 1.00 | 1.00 | 1.00 | 1.00 | 0.98(±0.04) |
| Sen | 1.00 | 1.00 | 1.00 | 1.00 | 1.00 | 1.00(±0.00) |
| Spe | 0.75 | 1.00 | 1.00 | 1.00 | 1.00 | 0.95(±0.10) |
| F1 | 0.91 | 1.00 | 1.00 | 1.00 | 1.00 | 0.98(±0.04) |
| AUC | 0.95 | 1.00 | 1.00 | 1.00 | 1.00 | 0.99(±0.02) |

**Supplementary table 5**

| 54 Structural Data + NAA/Cr in the frontal region | | | | | | |
| --- | --- | --- | --- | --- | --- | --- |
| Group | 1 | 2 | 3 | 4 | 5 | Mean(±Std) |
| ACC | 0.89 | 0.89 | 0.89 | 1.00 | 1.00 | 0.93(±0.05) |
| Sen | 1.00 | 1.00 | 1.00 | 1.00 | 1.00 | 1.00(±0.00) |
| Spe | 0.75 | 0.67 | 0.67 | 1.00 | 1.00 | 0.82(±0.15) |
| F1 | 0.91 | 0.92 | 0.92 | 1.00 | 1.00 | 0.95(±0.04) |
| AUC | 0.95 | 1.00 | 0.94 | 1.00 | 1.00 | 0.98(±0.03) |

**Supplementary table 6**

| 54 Structural Data + NAA/Cr in the parietal region | | | | | | |
| --- | --- | --- | --- | --- | --- | --- |
| Group | 1 | 2 | 3 | 4 | 5 | Mean(±Std) |
| ACC | 0.89 | 1.00 | 1.00 | 1.00 | 1.00 | 0.98(±0.04) |
| Sen | 1.00 | 1.00 | 1.00 | 1.00 | 1.00 | 1.00(±0.00) |
| Spe | 0.75 | 1.00 | 1.00 | 1.00 | 1.00 | 0.95(±0.10) |
| F1 | 0.91 | 1.00 | 1.00 | 1.00 | 1.00 | 0.98(±0.04) |
| AUC | 0.95 | 1.00 | 1.00 | 1.00 | 1.00 | 0.99(±0.02) |

**Supplementary table 7**

| 54 Structural Data + 3 Metabolic Data in the parietal region | | | | | | |
| --- | --- | --- | --- | --- | --- | --- |
| Group | 1 | 2 | 3 | 4 | 5 | Mean(±Std) |
| ACC | 1.00 | 1.00 | 1.00 | 1.00 | 1.00 | 1.00(±0.00) |
| Sen | 1.00 | 1.00 | 1.00 | 1.00 | 1.00 | 1.00(±0.00) |
| Spe | 1.00 | 1.00 | 1.00 | 1.00 | 1.00 | 1.00(±0.00) |
| F1 | 1.00 | 1.00 | 1.00 | 1.00 | 1.00 | 1.00(±0.00) |
| AUC | 1.00 | 1.00 | 1.00 | 1.00 | 1.00 | 1.00(±0.00) |

**Note:** Supplementary tables 1-7 correspond to the seven models in Table 3 respectively. ACC represents the accuracy of the model, Sen represents the sensitivity, SPE represents the specificity, F1 represents F1 score, and Group represents the group number of cross validation training. And we kept two decimal places in the result.
